# 

**Published:** 1881-12

**Authors:** 


### NECROLOGICAL.

Dr. T. Clay Maddux, a surgeon of some note in Baltimore, was shot and killed during an election affray, at Odenton, Md., on Tuesday, November 8th, 1881.

Dr. Benj. Franklin Bache, Medical Director, U. S. Navy, died at Brooklyn, on November 1st. He was 80 years of age, and had served as an officer of the Navy for nearly sixty years.

Dr. A. C. Holt, a native of Augusta, Ga., and for many years a popular practitioner of New Orleans, died at Summit, Miss., October 5th, 1881, aged 61 years.

Dr. John Menninger died in this city early in November. He was a native of Germany, and came to this country a political refugee in 1850.

Dr. A. Foster Axson died in New Orleans, September 12, aged 65 years. He was a native of Charleston, S. C., and graduated in 1836.

Dr. Frederick Horner died at Warrenton, Va., on the 18th of October, aged 76 years.

Dr. Jas. L. Pancoast, of Philadelphia, died at Long Branch, on September 29th. He was the eldest son of Dr. Wm. H. Pancoast.

Dr. John B. Brinton died at West Chester, Pa., on October 13th, at the age of 77 years.

### PROSPECTUS

**For Vol. III, 1882, of**

### The Independent Practitioner:

A MONTHLY JOURNAL,

DEVOTED TO

Medicine, Surgery, Obstetrics, Dentistry, Hygiene and Popular Science.

*This Journal is cosmopolitan in its circulation, management and scientific resources, and of equal interest to the specialist and general practitioner throughout the civilized world.*

*Its corps of editors and collaborators will represent Medicine, Surgery, Obstetrics, Dentistry, Hygiene and Popular Science, and in these we will seek for that which is new and useful, and give the refined findings to our readers. Politics, religious creeds and isms, or preferences in colleges and cliques will be excluded, and we will uphold truth and justice with independence.*

**Seven Reasons why every one interested in its Topics should subscribe now.**

*1st. It contains original papers from the best men in the profession.*

*2d. Its translations and selections contain more valuable and practical matter in an equal space than any other Journal published.*

*3d. Its Book Reviews are thorough criticisms made by competent writers, and in the interest of the profession.*

*4th. It treats Dentistry as a specialty of general Medicine and Surgery, and endeavors to strengthen the relationship between the different branches of Medical and Surgical Science and Art.*

*5th. It furnishes original and selected matter of a high scientific character in its Popular Science Department.*

*6th. It gives notes and items of interest, relating to medical and scientific progress throughout the world.*

*7th.* Each number contains 72 pages of reading matter, *printed on fine white super-calendered paper, with new type; compares favorably with any Medical Journal published, and its subscription price is within the reach of all progressive practitioners.*

### PERSONAL.

The following extracts COMMENDATORY OF THE INDEPENDENT PRACTITIONER are taken from a large number of letters, from which we could fill pages with similar expressions.

PROF. ISAAC E. TAYLOR, M. D., President of Bellevue Medical College, New York, says: "I have been much pleased with the matter introduced, as well as the dress of the Journal, and I wish you all success in the effort to advance the interests of the profession. I noticed a favorable criticism in the *Medical Record* of last week."

PROF. ROBERTS BARTHOLOW, M. D., of the Jefferson Medical College, Philadelphia, says: "I shall be pleased to send you a paper when I get through the work which I am compelled to do."

PROF. J. M. DA COSTA, M. D., of the same college and city: "I wish it (the Journal) a most marked success."

PROF. THOMAS H. CHANDLER, Dean of the Harvard University, Dental Department. "If you keep up the standard, your magazine will fill a void in our professional literature."

PROF. F. PEYRE PORCHER, M. D., Charleston, S. C.: "I have read your Journal with pleasure."

PROF. MIDDLETON MICHEL, M. D., of the same city and college: "I am much pleased with the appearance and contents of your Journal and wish it success."

PROF. JAS. E. GARRETSON, M. D., D. D. S., Dean of the Philadelphia Dental College: "Let me wish you success in an enterprise which looks as if calculated to be of great use to the dentist. I have read each number of the Journal with much satisfaction and have to apologize for my tardiness in acknowledging your kindness in sending it. I welcome the PRACTITIONER particularly from the standpoint of its *medico*-dental character. The more dentists learn of general medicine and surgery the better are they found qualified for work as specialists."

PROF. J. C. LE HARDY, M. D., of Savannah, Ga.: "I am very much pleased with the 'INDEPENDENT PRACTITIONER."

PROF. V. H. TALIAFERRO, M. D., of Atlanta, Ga., "I will cheerfully contribute my mite to aid in your effort to continue the Journal a grand success."

PROF. W. H. DOUGHTY, M. D., Augusta, Ga., "I wish your enterprise abundant success."

COL. J. J. WOODWARD, Surgeon U. S. Army, "I wish for your enterprise a useful and honorable career and all success."

DR. B. P. ANDERSON, President of the Medical Society of the State of Colorado: "Your Journal is a perfect success. It will give me a great deal of pleasure to assist the 'INDEPENDENT PRACTITIONER' in any way I can."

PROF. WM. A. HAMMOND, M. D., N. Y., and formerly Surgeon-General of the U. S. Army, "I am pleased with the 'INDEPENDENT PRACTITIONER,' and wish you success." Later (Oct. 30th, '81,) "I will ere long send you a paper which may prove of some interest to your readers."

PROF. ISAAC J. WETHERBEE, D. D. S., President of the Boston Dental College, "A careful examination of the contents of the 'INDEPENDENT PRACTITIONER' leads me to believe that it will be a very useful publication to the dental profession, and hope that you may meet with large success."

SAM. D. RAMBO, D. D. S., of Rio, Brazil: "I find the 'INDEPENDENT PRACTITIONER' very good. The best Journal out."

W. C. BARRETT, D.D.S., M. D., Buffalo, N. Y.: "The INDEPENDENT PRACTITIONER is a very good Journal indeed, and fills an important and otherwise vacant niche in our professional edifice."

DR. B. W. HARDER, of Savannah, Ga.: "Merit must and will meet with success."

Space will allow of the mention of a few more names only, of those who have sent us words of encouragement, or promised us original papers: PROF. AUSTIN FLINT, Sr., M. D., PROF. FRANK H. HAMILTON, M. D., PROF. T. GAILLARD THOMAS, M. D., ALFRED L. LOOMIS, M. D., J. MARION SIMS, M. D., GEO. M. BEARD, and others in our city; PROF. WM. PEPPER, M. D., PROF. S. WEIR MITCHELL, M. D., RICHARD J. DUNGLISON, M. D., and PROF. CHAS. J. ESSIG, D. D. S., M. D., of Philadelphia.

**PROSPECTUS OF VOL. II, 1881,**

*OF THE*

### Independent Practitioner:

### A MONTHLY JOURNAL.

*DEVOTED TO MEDICAL SURGICAL, OBSTETRICAL, DENTAL AND HYGIENICAL SCIENCE.*

---

The fixed purpose of the Editors of this journal is to adhere with unvarying fidelity to independence in all matters appertaining to its conduct and management It will know no sectionalism, and, therefore, confidently expects to merit the friendship and good will of those who *really* desire to sustain and keep from degradation to the level of the merest trade, the grandest and noblest calling known to humanity. From all such, subscriptions, contributions on subjects of interest to the profession, and advertisements are respectfully solicited. Our experience in the past, will be brought into activity in catering to the taste of its readers, and nothing will ever mar its pages not consistent with the standard that has been erected for its conduct and management. Being entirely unconnected with colleges and cliques, it will be bold, fearless and honest in speaking and upholding the right in *all* matters coming within its purview, and will scrupulously render even-handed justice to every one. All subjects of interest to Physicians, Obstetricians, Surgeons and Dentists will claim the attention of its editors and find space in its pages. To this end translations from the French and German periodicals will be introduced from time to time, and the English and American journals made tributary to its columns. Exchanges of Medical, Dental and other scientific journals are respectfully requested from all parts of the world. Exchanges and books, except Dental, for notice or review, should be addressed to the senior editor, and Dental exchanges, subscriptions, advertisements, and *all* matters relating to the business management of the journal, should be sent invariably to the junior editor and proprietor.

HARVEY L. BYRD, A. M., M. D.,

*No. 225 N. Gilmor Street.*

BASIL M. WILKERSON, D. D. S., M. D.,

*No. 68 N. Charles Street.*

### SPECIAL NOTICE.

From among a large number of letters commendatory of THE INDEPENDENT PRACTITIONER, we take pleasure in making excerpts from a few from different sections of our country, indicative of the estimate of our labors thus far.

SAM. D. RAMBO, D. D. S., of Rio, Brazil, says: I find the INDEPENDENT PRACTITIONER very good the best journal out.

PROF. ISAAC E. TAYLOR, M. D., President of Bellevue Medical College, New York, says "I have been much pleased with the matter introduced, as well as the dress of the Journal, and I wish you all success in the effort to advance the interests of the profession. I noticed a favorable criticism in the *Medical Record* of last week." A few weeks later he gave us a valuable paper which is published in our February issue.

PROF. ROBERTS BARTHOLOW, M. D., of the Jefferson Medical College, Philadelphia, says: "I trust the Journal is succeeding and that you find your editorial work congenial. I shall be pleased to send you a paper when I get through the work which I am compelled to do."

PROF. J. M. DA COSTA, M. D., of the same college and city, writes: "I wish it (the Journal) a most marked success."

PROF. THOS. H. CHANDLER, Dean of the Harvard University, Dental Department, writes: "I am well pleased with your first number. If you keep up the standard, your magazine will fill a void in our professional literature."

PROF. F. PEYRE PORCHER, M. D., Charleston, S. C., writes: "I have read your Journal with pleasure."

PROF. MIDDLETON MICHEL, M. D., of the same city and college, says: "I am much pleased with the appearance and contents of your Journal and wish it success."

PROF. JAS. E. GARRETSON, M. D., D. D. S., Dean of the Philadelphia Dental College, write "Let me wish you success in an enterprise which looks as if calculated to be of great use to the dentist. I have read each number of the Journal with much satisfaction and have to apologize for my tardiness in acknowledging your kindness in sending it. I welcome the 'PRACTITIONER' particularly from the standpoint of its *medico*-dental character. The more dentists learn of general medicine and surgery the better are they found qualified for work as specialists."

PROF. J. C. LE HARDY, M. D., of Savannah, Ga., writes: "I am very much pleased with the 'INDEPENDENT PRACTITIONER.'"

PROF. V. H. TALIAFERRO, M. D., of Atlanta, Ga., says: "I will cheerfully contribute my mite to aid in your effort to continue the Journal a grand success."

PROF. W. H. DOUGHTY, M. D., Augusta, Ga., "I wish your enterprise abundant success."

COL. J. J. WOODWARD, Surgeon U. S. Army, "I wish for your enterprise a useful and honorable career and all success."

DR. B. P. ANDERSON, President of the Medical Society of the State of Colorado: "Your Journal is a perfect success. It will give me a great deal of pleasure to assist the 'INDEPENDENT PRACTITIONER' in any way I can."

PROF. WM. A. HAMMOND, M. D., N. Y., and formerly Surgeon-General of the U. S. Army, says: "I am pleased with the 'INDEPENDENT PRACTITIONER,' and wish you success."

PROF. ISAAC J. WETHERBEE, D. D. S., President of the Boston Dental College, writes: "A careful examination of the contents of the 'INDEPENDENT PRACTITIONER' leads me to believe that it will be a very useful publication to the dental profession, and hope that you may meet with large success."

DR. W. R. MUNROE, of Baltimore, "I am happy to receive the 'INDEPENDENT PRACTITIONER,' so neatly and beautifully published, and so ably edited by yourself and colleague. Your prospectus is striking and convincing as to the propriety of your independent position, and I wish you great success in the enterprise you have so nobly inaugurated."

Others among the most intelligent members of the profession in this city have verbally expressed similarly encouraging words.

DR. B. W. HARDEE, of Savannah, Ga., "Merit must and will meet with success."

The foregoing extracts have been taken almost at random from a large number of letters, now before us, and pages could be filled with similar expressions from them.

Space will allow of the mention of a few more names only, of those who have sent us happy greetings or promised articles, viz.: PROF. WM. PEPPER, M. D., PROF. S. WEIR MITCHELL, M. D., DR RICHARD J. DUNGLINSON, PROF. CHAS. J. ESSIG, D. D. S, M. D., Philadelphia; PROF. T. GAILLARD THOMAS, M. D., DR. GEO. M. BEARD, and others, of New York, etc.

We hazard nothing in saying that no medical journal started in this country has been more generally commended, verbally and otherwise, by readers entitled to respect and confidence.

### SPECIAL NOTICE.

From among a large number of letters commendatory of THE INDEPENDENT PRACTITIONER, we take pleasure in making excerpts from a few from different sections of our country, indicative of the estimate of our labors thus far.

SAM. D. RAMBO, D. D. S., of Rio, Brazil, says I find the INDEPENDENT PRACTITIONER very good the best journal out.

The *Independent Practitioner* is a very good Journal indeed, and fills an important and otherwise vacant niche in our professional edifice, Very truly, &c., W. C. BARRETT.

PROF. ISAAC E. TAYLOR, M. D., President of Bellevue Medical College, New York, says "I have been much pleased with the matter introduced, as well as the dress of the Journal, and I wish you all success in the effort to advance the interests of the profession. I noticed a favorable criticism in the *Medical Record* of last week." A few weeks later he gave us a valuable paper which is published in our February issue.

PROF. ROBERTS BARTHOLOW, M. D., of the Jefferson Medical College, Philadelphia, says: "I trust the Journal is succeeding and that you find your editorial work congenial. I shall be pleased to send you a paper when I get through the work which I am compelled to do."

PROF. J. M. DA COSTA, M. D., of the same college and city, writes: "I wish it (the Journal) a most marked success."

PROF. THOS. H. CHANDLER, Dean of the Harvard University, Dental Department, writes: "I am well pleased with your first number. If you keep up the standard, your magazine will fill a void in our professional literature."

PROF. F. PEYRE PORCHER, M. D., Charleston, S. C., writes: "I have read your Journal with pleasure."

PROF. MIDDLETON MICHEL, M. D., of the same city and college, says: "I am much pleased with the appearance and contents of your Journal and wish it success."

PROF. JAS. E. GARRETSON, M. D., D. D. S., Dean of the Philadelphia Dental College, writes "Let me wish you success in an enterprise which looks as if calculated to be of great use to the dentist. I have read each number of the Journal with much satisfaction and have to apologize for my tardiness in acknowledging your kindness in sending it. I welcome the 'PRACTITIONER' particularly from the standpoint of its *medico*-dental character. The more dentists learn of general medicine and surgery the better are they found qualified for work as specialists."

PROF. J. C. LE HARDY, M. D., of Savannah, Ga., writes: "I am very much pleased with the 'INDEPENDENT PRACTITIONER.'"

PROF. V. H. TALIAFERRO, M. D., of Atlanta, Ga., says: "I will cheerfully contribute my mite to aid in your effort to continue the Journal a grand success."

PROF. W. H. DOUGHTY, M. D., Augusta, Ga., "I wish your enterprise abundant success."

COL. J. J. WOODWARD, Surgeon U. S. Army, "I wish for your enterprise a useful and honorable career and all success."

DR. B. P. ANDERSON, President of the Medical Society of the State of Colorado: "Your Journal is a perfect success. It will give me a great deal of pleasure to assist the 'INDEPENDENT PRACTITIONER' in any way I can."

PROF. WM. A. HAMMOND, M. D., N. Y., and formerly Surgeon-General of the U. S. Army, says: "I am pleased with the 'INDEPENDENT PRACTITIONER,' and wish you success."

PROF. ISAAC J. WETHERBEE, D. D. S., President of the Boston Dental College, writes: "A careful examination of the contents of the 'INDEPENDENT PRACTITIONER' leads me to believe that it will be a very useful publication to the dental profession, and hope that you may meet with large success."

DR. W. R. MUNROE, of Baltimore, "I am happy to receive the 'INDEPENDENT PRACTITIONER,' so neatly and beautifully published, and so ably edited by yourself and colleague. Your prospectus is striking and convincing as to the propriety of your independent position, and I wish you great success in the enterprise you have so nobly inaugurated."

Others among the most intelligent members of the profession in this city have verbally expressed similarly encouraging words.

DR. B. W. HARDEE, of Savannah, Ga., "Merit must and will meet with success."

The foregoing extracts have been taken almost at random from a large number of letters, now before us, and pages could be filled with similar expressions from them.

Space will allow of the mention of a few more names only, of those who have sent us happy greetings or promised articles, viz.: PROF. WM. PEPPER, M. D., PROF. S. WEIR MITCHELL, M. D., DR. RICHARD J. DUNGLINSON, PROF. CHAS. J. ESSIG, D. D. S., M. D., Philadelphia; PROF. T. GAILLARD THOMAS, M. D., DR. GEO. M. BEARD, and others, of New York, etc.

We hazard nothing in saying that no medical journal started in this country has been more generally commended, verbally and otherwise, by readers entitled to respect and confidence.

### PERSONAL.

*68 North Charles Street, Baltimore, Md., January 1st, 1881.*

DEAR DOCTOR:—We take pleasure in sending you this Sample Copy of THE INDEPENDENT PRACTITIONER, and hope that you may find it a journal worthy of your support.

Our contributors and subscribers rank among the ablest members of the professions. With the aid of your pen and means we hope to make it one of the best professional periodicals of this country.

*Should we not hear from you by the 1st of February, inst., we will consider that you have kindly consented to become one of our subscribers, and will, with pleasure, place your name on our subscription list. If it does not suit you to subscribe you will please notify us by postal card at your earliest opportunity.*

Each Number will contain fifty pages of reading matter, making an Annual Volume of 600 pages for $2.00

### Special Inducements to Cash Subscribers.

We have made arrangements so that our cash subscribers can secure, through us, any of the following publicaiions at the "net" rates quoted below. By taking our journal with others the price of THE INDEPENDENT PRACTITIONER can be saved, also the trouble and risk of sending money and orders to several publishers.

Should Our Subscribers desire any Magazine or Paper which is not on the list, we will have it mailed regularly to them by the publishers at clubbing rates to us.

| | Regular (Regular Subscription Rates.) | Net. (Rates to $2.00 Cash Subscribers to "The Independent Practitioner.") |
|---|---|---|
| Scientific American | $ 3 20 | $ 2 56 |
| " Supplement | 5 00 | 4 00 |
| Popular Science Monthly | 5 00 | 4 00 |
| Appleton's Journal | 3 00 | 2 25 |
| N. American Review | 5 00 | 3 75 |
| New York Medical Journal | 4 00 | 3 00 |
| Lippincott's Magazine | 3 00 | 2 25 |
| Philadelphia Medical Times | 4 00 | 3 00 |
| Archives of Dermatology | 4 00 | 3 00 |
| Harper's Magazine | 4 00 | 3 00 |
| " Weekly | 4 00 | 3 20 |
| " Bazaar | 4 00 | 3 20 |
| " Young People | —— | 1 20 |
| Scribner's Monthly | 4 00 | 3 20 |
| St. Nicholas | 3 00 | 2 40 |
| Frank Leslie's Illus. Paper | 4 00 | 3 00 |
| " " Chimney Corner | 4 00 | 3 00 |
| " " Ladies' Journal | 4 00 | 3 00 |
| " " Boys and Girls | 2 50 | 1 75 |
| " " Ladies' Magazine | 3 50 | 2 75 |
| " " Sunday Magazine | 3 00 | 2 25 |

| | Regular (Regular Subscription Rates.) | Net. (Rates to $2.00 Cash Subscribers to "The Independent Practitioner.") |
|---|---|---|
| Frank Leslie's Popular Mon. | $ 3 00 | $2 25 |
| " " Pleasant Hours | 1 50 | 1 00 |
| " " Budget | 2 00 | 1 50 |
| Demorest's Monthly Mag'ne | 3 00 | 1 80 |
| The Atlantic Monthly | —— | 3 20 |
| Detroit Free Press, Weekly. | 2 00 | 1 25 |
| Baltimore Gazette, Daily | 6 00 | 4 80 |
| N. Y. Tribune, Weekly | —— | 1 00 |
| " " Semi-Weekly. | —— | 2 00 |
| " World, Daily, with Sunday Edition | 12 00 | 9 60 |
| " World, Daily, without Sunday Edition | 10 00 | 8 00 |
| " World, Semi-Weekly | 2 00 | 1 60 |
| " World, Weekly | 1 00 | 80 |
| " Sun, Daily | 6 50 | 5 85 |
| " Sun, Sunday Edition | 1 20 | 1 08 |
| " Snn, Weekly | 1 00 | 90 |
| " Times, Semi-Weekly | 2 50 | 2 25 |
| " Times, Weekly | 1 00 | 90 |
| Baltimore Eve'g News, d'ly. | 6 00 | 4 80 |

**ADD $2.00 FOR THE INDEPENDENT PRACTITIONER.**

WE WILL SEND FOR CASH

### Webster's New Edition Unabridged Dictionary,

Sheep, Marble Edges, Price $12.00, with "The Independent Practitioner" for 1881 for $12.00. Or Webster's National Pictorial Dictionary, Sheep Binding, Price $5.00, with "The Independent Practitioner" for $6.00. Freight on Dictionary to be collected on delivery.

To secure the above advantages cash must accompany each order with annual subscriptions for 1881 on each periodical.

Please fill attached subscription blank and remit by Post Office Money Order, Registered Letter, or Check payable in Banks at New York or Baltimore.

Fraternally yours,

B. M. WILKERSON, *Proprietor.*

### SPECIAL NOTICE.

From among a large number of letters commendatory of THE INDEPENDENT PRACTITIONER, we take pleasure in making excerpts from a few from different sections of our country, indicative of the estimate of our labors thus far.

SAM. D. RAMBO, D. D. S., of Rio, Brazil, says I find the INDEPENDENT PRACTITIONER very good. The best journal out.

The *Independent Practitioner* is a very good Journal indeed, and fills an important and otherwise vacant niche in our professional edifice. Very Truly, &c., W. C. BARRETT.

PROF. ISAAC E. TAYLOR, M. D., President of Bellevue Medical College, New York, says "I have been much pleased with the matter introduced, as well as the dress of the Journal, and I wish you all success in the effort to advance the interests of the profession. I noticed a favorable criticism in the *Medical Record* of last week." A few weeks later he gave us a valuable paper which is published in our February issue.

PROF. ROBERTS BARTHOLOW, M. D., of the Jefferson Medical College, Philadelphia, says: "I trust the Journal is succeeding and that you find your editorial work congenial. I shall be pleased to send you a paper when I get through the work which I am compelled to do."

PROF. J. M. DA COSTA, M. D., of the same college and city, writes: "I wish it (the Journal) a most marked success."

PROF. THOS. H. CHANDLER, Dean of the Harvard University, Dental Department, writes: "I am well pleased with your first number. If you keep up the standard, your magazine will fill a void in our professional literature."

PROF. F. PEYRE PORCHER, M. D., Charleston, S. C., writes: "I have read your Journal with pleasure."

PROF. MIDDLETON MICHEL, M. D., of the same city and college, says: "I am much pleased with the appearance and contents of your Journal and wish it success."

PROF. JAS. E. GARRETSON, M. D., D. D. S., Dean of the Philadelphia Dental College, write "Let me wish you success in an enterprise which looks as if calculated to be of great use to the dentist. I have read each number of the Journal with much satisfaction and have to apologize for my tardiness in acknowledging your kindness in sending it. I welcome the 'PRACTITIONER' particularly from the standpoint of its *medico*-dental character. The more dentists learn of general medicine and surgery the better are they found qualified for work as specialists."

PROF. J. C. LE HARDY, M. D., of Savannah, Ga., writes: "I am very much pleased with the 'INDEPENDENT PRACTITIONER.'"

PROF. V. H. TALIAFERRO, M. D., of Atlanta, Ga., says: "I will cheerfully contribute my mite to aid in your effort to continue the Journal a grand success."

PROF. W. H. DOUGHTY, M. D., Augusta, Ga., "I wish your enterprise abundant success."

COL. J. J. WOODWARD, Surgeon U. S. Army, "I wish for your enterprise a useful and honorable career and all success."

DR. B. P. ANDERSON, President of the Medical Society of the State of Colorado: "Your Journal is a perfect success. It will give me a great deal of pleasure to assist the 'INDEPENDENT PRACTITIONER' in any way I can."

PROF. WM. A. HAMMOND, M. D., N. Y., and formerly Surgeon-General of the U. S. Army, says: "I am pleased with the 'INDEPENDENT PRACTITIONER,' and wish you success."

PROF. ISAAC J. WETHERBEE, D. D. S., President of the Boston Dental College, writes: "A careful examination of the contents of the 'INDEPENDENT PRACTITIONER' leads me to believe that it will be a very useful publication to the dental profession, and hope that you may meet with large success."

DR. W. R. MUNROE, of Baltimore, "I am happy to receive the 'INDEPENDENT PRACTITIONER,' so neatly and beautifully published, and so ably edited by yourself and colleague. Your prospectus is striking and convincing as to the propriety of your independent position, and I wish you great success in the enterprise you have so nobly inaugurated."

Others among the most intelligent members of the profession in this city have verbally expressed similarly encouraging words.

DR. B. W. HARDEE, of Savannah, Ga., "Merit must and will meet with success."

The foregoing extracts have been taken almost at random from a large number of letters, now before us, and pages could be filled with similar expressions from them.

Space will allow of the mention of a few more names only, of those who have sent us happy greetings or promised articles, viz.: PROF. WM. PEPPER, M. D., PROF. S. WEIR MITCHELL, M. D., DR RICHARD J. DUNGLINSON, PROF. CHAS. J. ESSIG, D. D. S, M. D., Philadelphia; PROF. T. GAILLARD THOMAS, M. D., DR. GEO. M. BEARD, and others, of New York, etc.

We hazard nothing in saying that no medical journal started in this country has been more generally commended, verbally and otherwise, by readers entitled to respect and confidence.

WILL *YOU* PLEASE SEND IN *YOUR* SUBSCRIPTION MONEY *NOW?*

Money is at OUR RISK ONLY when sent by Post Office Money Order, Registered Letter, or Check on Banks in New York City, made payable to INDEPENDENT PRACTITIONER, 20 Courtlandt street, N. Y. City.

If you prefer to send check on your private bank, unless located in the city of New York, please add 25 cents for collection.

